# Remodelling hierarchical NiCo_2_O_4_@ZnS nanorods with multi-walled carbon nanotubes as a counter electrode for dye-sensitized solar cell applications

**DOI:** 10.1038/s41598-026-38255-7

**Published:** 2026-02-01

**Authors:** Methawee Nukunudompanich, Theeranuch Nachaithong, Phatcharin Phumuen, Wassana Wannabut, Neeraphat Kunbuala, Supinya Nijpanich, Kongsak Pattarith, Yonrapach Areerob

**Affiliations:** 1 Department of Industrial Engineering, School of Engineering, King Mongkut’s Institute of Technology Ladkrabang, Bangkok, 10520 Thailand; 2 Institute for Integrated Radiation and Nuclear Science, Kyoto University, 2-1010, Asahiro Nishi, Kumatori, Sennan-gun, Osaka, 590-0494 Japan; 3 Department of Physics, Faculty of Science, Khon Kaen University, Khon Kaen, 40002 Thailand; 4 Department of Biomedical Engineering, Tohoku University, Sendai, 980-8579 Japan; 5 Synchrotron Light Research Institute (Public Organization), 111 University Avenue, Muang District, Nakhon Ratchasima, 30000 Thailand; 6 Department of Chemistry, Faculty of Science, Buriram Rajabhat University, Buriram, 31000 Thailand

**Keywords:** NiCo_2_O_4_@ZnS/MWCNT nanocomposite, Counter electrodes, Dye-sensitized solar cells, Chemistry, Energy science and technology, Materials science, Nanoscience and technology

## Abstract

A hierarchical NiCo_2_O_4_@ZnS/MWCNT (NCO@Z-MWCNTs) nanocomposite was synthesized to serve as a platinum-free counter electrode for dye-sensitized solar cells (DSSCs). The nanocomposite comprised spinel NiCo_2_O_4_ nanorods, ZnS associated with the surface of the nanorods, and an interconnected multi-walled carbon nanotube (MWCNT) network, and it was synthesized via a low-temperature solution-based hydrothermal method. XRD confirmed the presence of cubic NiCo_2_O_4_ and zinc blende ZnS phases, while FESEM–EDS and XPS analyses verified the incorporation of ZnS and the formation of a conductive carbon framework interconnecting adjacent nanorods. ZnS, rather than acting as an isolated catalytic component, was considered to contribute additional sulfide-related surface sites and to modulate the interfacial electronic environment of the NiCo_2_O_4_ nanorods, which likely facilitated redox reactions involving the I^−^/I_3_^−^ couple. Meanwhile, the MWCNT network established continuous electron transport pathways, effectively reducing interfacial resistance and enhancing charge-transfer efficiency. Thermogravimetric and electrochemical analyses revealed enhanced thermal stability, improved redox kinetics, and a significant reduction in charge-transfer resistance compared with pristine NiCo_2_O_4_.The optimized NCO@Z–MWCNT 9wt% counter electrode achieved a power conversion efficiency of 10.03% under AM 1.5 G illumination, exceeding that of the Pt reference device (9.6%). Overall, the improved performance was attributed to the combined contributions of ZnS surface modification and the conductive MWCNT network, which together enhanced charge transport and electrocatalytic activity. This work demonstrates a scalable strategy for developing cost-effective, durable, and high-performance counter electrodes for dye-sensitized solar cells.

**Supplementary Information:**

The online version contains supplementary material available at 10.1038/s41598-026-38255-7.

## Introduction

 A sustained rise in energy demand, combined with environmental pollution and the finite supply of fossil fuels, is accelerating the transition toward renewable energy systems. Solar energy is becoming a prime renewable energy option as performance improves and costs decrease. Among photovoltaic cells, dye-sensitized solar cells (DSSCs) generate current by dye-mediated electron injection into a mesoporous layer and can be fabricated by low-temperature, scalable processes. In a DSSC, a surface-anchored photosensitizer absorbs photons and reaches an excited state; electrons are injected into the conduction band of a wide-bandgap semiconductor (typically a mesoporous TiO_2_ film on a transparent conducting oxide)^1^. The oxidized dye is then reduced by an iodide/triiodide (I^−^/I_3_^−^) redox mediator, which carries charge through the electrolyte to complete the catalytic cycle at the counter electrode. A standard device comprises a dye-sensitized TiO_2_ photoanode on FTO or ITO, a liquid I^−^/I_3_^−^ electrolyte, and a counter electrode (CE). The CE is typically composed of platinum (Pt), which is chosen for its high conductivity and electrochemical stability^2^. Pt as the CE collects electrons from the external circuit and catalyzes the reduction of I_3_^−^ to I^−^; its charge-transfer kinetics and series resistance strongly affect the fill factor and overall efficiency^3^. Pt has low overpotential and high conductivity, but its cost and supply issues, as well as its chemical vulnerability in iodide electrolytes (for example, the formation of PtI_4_ and surface poisoning by adsorbates), make it less durable and harder to scale up ^4,]^.

Platinum-free counter electrodes have therefore been actively explored. Candidates include transition-metal sulfides (NiS^6^, CuS^7^, MoS_2_^8^, WS_2_^9^), conductive carbons (graphene, multi-walled carbon nanotubes), MIn_2_S_4_ (M = Fe, Ni, Co), CoFeO_4_^10^, and mixed nickel–cobalt oxides/sulfides (NiCo_2_O_4_^11^, NiCo_2_S_4_^12^). These materials combine earth-abundant elements with good electrical conductivity, large accessible surface area, and numerous electrochemically active sites, yielding low charge-transfer resistance for the I_3_^−^/I^−^ couple^12,13^. These properties enable efficient triiodide (I_3_^−^) reduction, which is essential for improving charge transfer and overall device efficiency in DSSCs.

Nickel cobalt oxide (NiCo_2_O_4_, NCO) has gained attention as a Pt-free counter-electrode for DSSCs because its spinel structure offers abundant Co^3+^/Ni^3+^ redox sites for I_3_^−^ reduction and higher electrical conductivity than single-phase NiO or Co_3_O_4_^14^. NiCo_2_O_4_ can be synthesized by low-temperature solution-based processing techniques into films and diverse nanostructures (nanorods, nanoflowers, nanosheets, mesoporous networks)^14,15^ and is stable in I^−^/I_3_^−^ electrolytes. Its mesoporous architecture provides a large active surface area and facilitates ion transport, thereby improving charge-transfer kinetics and device performance. However, in prior work, a DSSC incorporating an NCO nanosheet-based CE delivered lower efficiency (6.84%) than did a corresponding device using an NCO nanoflower-based CE (8.48%), the latter’s performance being comparable to that of a Pt CE (8.11%)^16^. This highlighted the slower interfacial kinetics and higher resistance of the NCO rods. Moreover, while nanorods offer directional charge transport, their limited electroactive area and poor inter-rod percolation decrease charge transfer. To translate the ordered-nanorod advantage into higher efficiency, prior studies showed that an ultrathin shell could increase catalytic-site density, passivate surface defects, and accelerate interfacial charge transfer^17^, while a carbon-nanotube network restored lateral conductivity and reduced series resistance (R_s_) and shunt resistance (R_ct_). Among common shells (ZnO, TiO_2_, ZnS), Chang et al.^18^ reported NCO@ZnS core–shell nanocomposites supplied S-rich active sites for the I_3_^−^ → I^−^ reaction, and protected the oxide core in I^−^/I_3_^−^ electrolyte, thereby improving stability and charge-transfer kinetics. Carbon materials such as carbon black, CNTs, and graphene have been widely explored for use in Pt-free CEs due to their high conductivity and corrosion resistance^19,20^. In particular, multi-walled carbon nanotubes (MWCNTs) provided high surface area and a percolating conduction pathway that bridged adjacent rods and coupled them to the current collector^8,20,21^. In this framework, a ZnS shell together with an MWCNT network was designed to improve the performance of a DSSC with NCO nanorods as the CE.

In this work, pure NCO, NCO@ZnS, and NCO@ZnS integrated with MWCNT (NCO@Z–MWCNT) at loadings of 3 wt%, 5 wt%, 7 wt%, and 9 wt% were systematically evaluated as Pt-free counter electrodes. The integration of ZnS and an MWCNT network with NCO nanorods resulted in a maximum power conversion efficiency of 10.03% under AM 1.5G illumination, accompanied by improved charge-transfer behavior and reduced resistive losses. Rather than indicating a fundamentally new mechanism, the performance enhancement was attributed to the optimized hierarchical architecture, in which ZnS surface modification and the conductive MWCNT network collectively improved interfacial charge transfer and electron transport. The optimized NCO@Z–MWCNT (9%) electrode effectively exploits the ordered nanorod geometry to achieve competitive device-level performance with reduced resistance and enhanced charge-transfer kinetics.

## Experiment

### Chemical and materials

All chemicals and supplies were utilized as received, without further purification. Sigma-Aldrich supplied the MWCNTs, which were used as received in the preparation of the NCO@Z-MWCNT nanocomposite. The MWCNTs were obtained as a commercial product with CAS No. 308068-56-6 (> 95% carbon basis, diameter 50–90 nm), as specified by the supplier on the product page. Sigma-Aldrich also provided the medium molecular weight chitosan with a degree of deacetylation exceeding 75%, together with glacial acetic acid (99.7%). Furthermore, natural graphite powder (99.9%, < 45 μm), sodium nitrate (≥ 99%), sulfuric acid (98%), potassium permanganate (≥ 99%), and hydrogen peroxide solution (30% w/w in H_2_O) were obtained from Merck Chemicals. Nickel (II) nitrate hexahydrate (Ni(NO_3_)_2_·6 H_2_O, 98%), ammonium molybdate tetrahydrate ((NH_4_)_6_Mo_7_O_2__4_·4 H_2_O, ≥ 99%), and thiourea (≥ 99%) were obtained from Thermo Fisher Scientific for the manufacture of NiMoS_2_. The gamma irradiation process employed a cobalt-60 source at a dedicated facility (Nordion International Inc.), with samples housed in high-density polyethylene vials.

### Characterization

The NCO@Z-MWCNT counter electrodes were studied to ascertain their structural, morphological, and electrochemical properties. A Rigaku Miniflex 600 diffractometer utilizing Cu-Kα radiation (λ = 1.5406 Å) was employed to examine the crystalline structure of the NCO@Z-MWCNT electrodes. X-ray photoelectron spectroscopy (XPS) (PHI 5000 VersaProbe III, ULVAC-PHI) was used to analyze the elemental composition and oxidation states of Ni, Mo, S, C, and O in the composite under ultra-high vacuum conditions. Scanning electron microscopy (SEM) (JEOL JSM-7600 F) at 5 kV and transmission electron microscopy (TEM) (FEI Tecnai G2 F20) at 200 kV yielded high-resolution images of the morphology and surface characteristics of the NCO@Z-MWCNT composites. NCO nanoparticles were seen to be uniformly dispersed across the MWCNT in SEM images, whereas the multilayer architecture of the MWCNT and its incorporation with gamma-irradiated chitosan were confirmed in the TEM images. Brunauer-Emmett-Teller (BET) analysis conducted with a Micromeritics ASAP 2020 analyzer revealed an enhanced surface area of the composite, indicating its appropriateness for catalytic applications.

A total of seven counter electrodes were electrochemically assessed using a Metrohm Autolab PGSTAT204 workstation. The electrodes were the: (1) pure NCO, (2) NCO@Z, and the (3–7) NCO@Z–MWCNT composites with 1 wt%, 3 wt%, 5 wt%, 7 wt%, and 9 wt% MWCNT. The catalytic activity of each electrode was assessed via 100 mV/s cyclic voltammetry (CV) in an iodide/triiodide electrolyte solution. Electrochemical impedance spectroscopy (EIS) was performed at frequencies ranging from 10^−2^ to 10^5^ Hz with a bias potential of 10 mV. Nyquist plots were analyzed to ascertain charge transfer resistance (R_ct_) and diffusion resistance. The NCO@Z–MWCNT counter electrodes with 1 wt%, 3 wt%, 5 wt%, 7 wt%, and 9 wt% MWCNT loadings were incorporated into dye-sensitized solar cell (DSSC) devices to evaluate their photovoltaic performance. Testing was carried out under simulated sunlight using a solar simulator (Newport 94023 A, AM 1.5, 100 mW/cm^2^). A Keithley 2400 source meter was used to evaluate current density, voltage, fill factor (FF), and power conversion efficiency (PCE). The DSSC employing NCO@Z–MWCNT (9 wt%) as the counter electrode outperformed the platinum-based electrode, owing to the synergistic effects of NiCo_2_O_4_, ZnS, and MWCNT. This comprehensive analysis demonstrates that NCO@Z–MWCNT (9 wt%) is a promising and robust alternative counter electrode for DSSCs.

### Synthesis of hierarchical NCO, NCO@Z, and NCO@Z–MWCNT composites

Porous NCO nanostructures were synthesized via a hydrothermal method followed by thermal treatment. Initially, solution A was prepared by dissolving 1 mmol of Ni(NO_3_)_2_·6 H_2_O in 15 mL of deionized water under magnetic stirring for 30 min. In parallel, solution B was formulated by dissolving 0.2 g of Na_2_SO_4_, 1 mmol of citric acid, 1 mmol of Co(NO_3_)_2_·6 H_2_O, and 1 mmol of NH_4_HCO_3_ in 15 mL of deionized water with continuous stirring for 30 min. Solution B was then added dropwise into solution A and stirred for an additional 30 min to ensure homogeneous mixing. The resulting solution was transferred into a 35 mL Teflon-lined stainless-steel autoclave and subjected to hydrothermal treatment at 160 °C for 8 h. After naturally cooling to room temperature, the precipitate was collected by centrifugation, washed thoroughly with ethanol, and dried at 70 °C for 2 h. The dried precursor was subsequently calcined in air at 500 °C for 3 h to obtain porous NCO nanostructures with a nanorod-dominated hierarchical morphology.

The NiCo_2_O_4_@ZnS (NCO@Z) nanostructures were prepared through a secondary hydrothermal deposition process. Specifically, 10 g of the as-prepared NiCo_2_O_4_ powder was dispersed in 15 mL of deionized water and sonicated for 3 h to form a uniform suspension. Subsequently, 40 mL of 1 mM zinc acetate dihydrate (Zn(Ac)_2_·2 H_2_O) solution and thiourea (90 mM) were added to the suspension under continuous stirring for 30 min at ambient temperature. The resulting mixture was transferred into a 200 mL Teflon-lined stainless steel autoclave and maintained at 125 °C for 24 h. After completion of the reaction, the autoclave was allowed to cool naturally to room temperature. The resulting NCO@Z precipitate was collected by centrifugation, washed repeatedly with deionized water and ethanol to remove residual reactants, and dried at 70 °C for 3 h.

To fabricate NCO@Z–MWCNT composites with different MWCNT loadings, 10 g of the NCO@Z material was first dispersed in 15 mL of deionized water and magnetically stirred for 30 min at room temperature. Predetermined amounts of the multi-walled carbon nanotubes (MWCNTs) were then introduced into the suspension to obtain final MWCNT contents of 1, 3, 5, 7, and 9 wt% relative to the total composite mass. The corresponding masses of MWCNTs were 0.10, 0.30, 0.50, 0.70, and 0.90 g, respectively, while maintaining the NCO@Z host material at a fixed amount of 10 g for all samples. The mixture was stirred for 15 min to ensure uniform dispersion of the MWCNTs, after which an aqueous NaBH_4_ solution (0.05 M) was rapidly added to promote interfacial interaction and stabilization of the hybrid structure. The reaction was allowed to proceed under continuous stirring for an additional 30 min. The resulting composites were collected by centrifugation, thoroughly washed with deionized water and ethanol, and dried at 70 °C for 6 h. All NCO@Z–MWCNT composites were synthesized following the same procedure, with only the MWCNT loading varied to systematically investigate its influence on the structural, electrochemical, and photovoltaic properties.

### Fabrication of DSSCs

The FTO glass plates were subjected to three 15 min cycles of ultrasonication, followed by drying. Ethyl alcohol was used as both a solvent and dispersant to facilitate the uniform mixing and slurry formation of the NCO@Z–MWCNT active material. Each prepared colloid prepared was applied to FTO plates and allowed to dry for 12 h. To prepare a 5 mM solution, 0.208 g of H_2_PtCl_6_ was dissolved in 100 mL of isopropanol or ethanol. Subsequently, H_2_PtCl_6_ was applied to each FTO glass plate to produce FTO-Pt, and the assembly was heated to 450 °C for 30 min. The experiment employed the doctor-blading technique to apply a bilayer of TiO_2_ sol onto the FTO substrates. The substrates were subsequently heated at varying temperatures to fabricate the photoanodes. The TiO_2_ photoanodes were subsequently immersed in a 0.4 mM N719 acetonitrile solution for 24 h. The DSSCs were assembled in a sandwich-type configuration using N719-sensitized photoanodes, and their photovoltaic performance was evaluated under standard illumination conditions. A Surlyn film encapsulated the DSSCs, and an iodine-based electrolyte was utilized to fill them^21^.

## Results and discussion

### XRD analysis

**Fig. 1:**
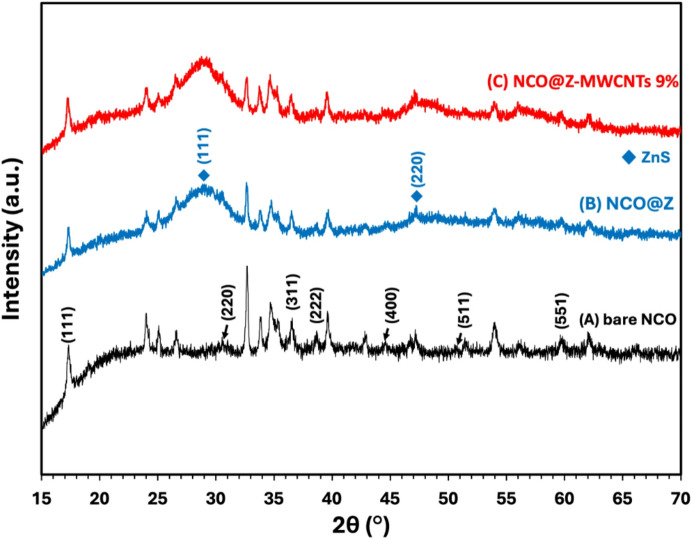
XRD patterns of (**A**) Pure NCO, (**B**) NCO@Z, and (**C**) NCO@Z-MWCNT 9 wt%.

**Fig. 2:**
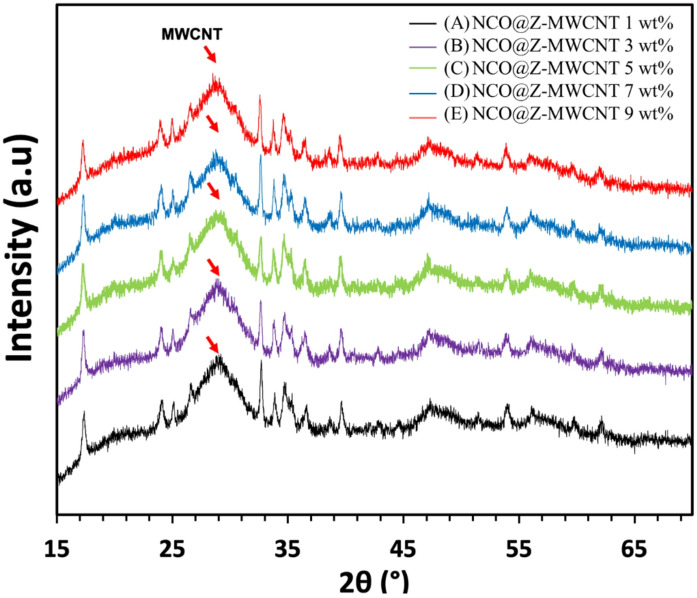
XRD patterns of NCO@Z-MWCNT composites with MWCNT loadings of 1 wt%, 3 wt%, 5 wt%, 7 wt%, and 9 wt%. (Red arrow indicates the broad graphitic feature of MWCNTs.)

Figure 1 shows the X-ray diffraction patterns of (A) NCO, (B) NCO@Z, and (C) NCO@Z–MWCNT (9 wt%). The diffraction peaks of sample (A) were very close to the standard ICDD reference code 00-020-0781 and 01-073-1704 spectra for nickel cobalt oxide. The sample showed characteristic peaks at 18.907°, 31.149°, 36.697°, 38.405°, 44.623°, and 50.477°, which corresponded to the (1 1 1), (2 2 0), (3 1 1), (2 2 2), (4 0 0), (5 1 1), (4 4 0), and (4 4 4) diffraction planes. The peaks validated the face-centered cubic (FCC) spinel configuration of NiCo_2_O_4_. In (B), NCO@Z, the characteristic NiCo_2_O_4_ peaks were still present, while new reflections at approximately 28.5°, 47.4°, and 56.3° emerged, which corresponded to the (111), (220), and (311) planes of cubic ZnS (zinc blende; e.g., ICDD code 01-089-2155 spectra), confirming the successful incorporation of crystalline ZnS into the composite. Subsequent analysis of sample (C) identified peaks corresponding to the reference codes 00-020-0781 and 01-073-1704 spectra for NiCo_2_O_4_, in addition to peaks associated with ZnS (ICDD code 01-089-2155). The crystal structures of NiCo_2_O_4_ and ZnS maintained their respective FCC configurations, indicating satisfactory material integration without phase transition. A weak broadband near 26° corresponded to graphitic structures (002), indicating the inclusion of MWCNT. Figure 2 presents the XRD comparison patterns of NCO@Z-MWCNT at different loadings: (a) 1 wt%, (b) 3 wt%, (c) 5 wt%, (d) 7wt%, and (e) 9 wt%. The samples exhibit diffraction peaks corresponding to the NiCo_2_O_4_ (ICDD codes 00-020-0781 and 01-073-1704) spectra and ZnS (ICDD code 01-089-2155) spectra, confirming the nature of the crystalline phases. Similarly, samples (a–e) exhibit characteristic peaks matching both reference codes, affirming the formation of the intended composite materials. Across the 1–9 wt% MWCNT variants, a broad feature near 26° appears and grows slightly with loading, attributable to the graphitic (002) band of the nanotubes. It does not introduce extra crystalline phases or obscure the diagnostic NiCo_2_O_4_ and ZnS lines.

### SEM-based EDS and TEM analysis

**Fig. 3:**
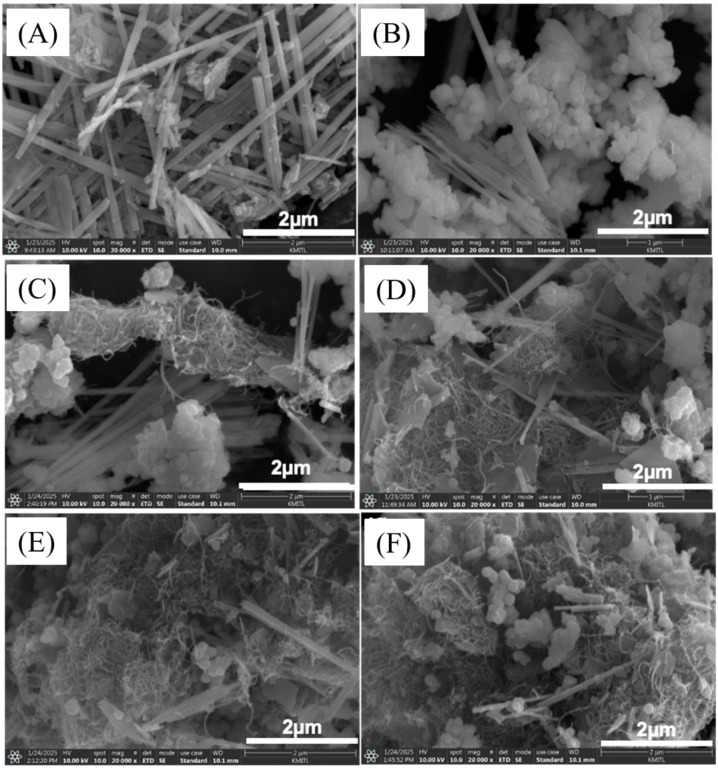
SEM images showing the morphology evolution of (**A**) pure NCO, (**B**) NCO@Z, (**C**) NCO@Z–MWCNT 3 wt%, (**D**) NCO@Z–MWCNT 5 wt%, (**E**) NCO@Z–MWCNT 7 wt%, and (**F**) NCO@Z–MWCNT 9 wt%. A predominantly one-dimensional nanorod-like framework is observed in all samples.

The morphological properties of the synthesized NCO, NCO@Z, and NCO@Z–MWCNT (with MWCNT contents of 3–9 wt%) samples were investigated using FESEM. The FESEM images indicate that pristine NCO forms straight one-dimensional nanorod-like structures, which are favorable for ion diffusion and electron transport. As shown in Fig. 3a, pure NCO consists of radially aligned nanorods with lengths exceeding 2 μm. Upon the introduction of ZnS, ZnS nanoparticles are uniformly deposited on the surface of the NCO nanorods, forming a ZnS-decorated nanorod architecture, rather than a classical core–shell structure with a continuous shell layer. As observed in Fig. 3b, ZnS appears as aggregated particle domains surrounding the NCO framework. Fig. 3c shows the sample with the lowest MWCNT content, where worm-like MWCNTs bridge neighboring ZnS-decorated nanorods, establishing conductive pathways between adjacent structures. With increasing MWCNT content (Figs. 3d–f), a progressive accumulation of MWCNT networks is observed, leading to more extensive coverage of the NCO@Z surface and the formation of a hierarchical nanorod–MWCNT composite architecture.

**Fig. 4:**
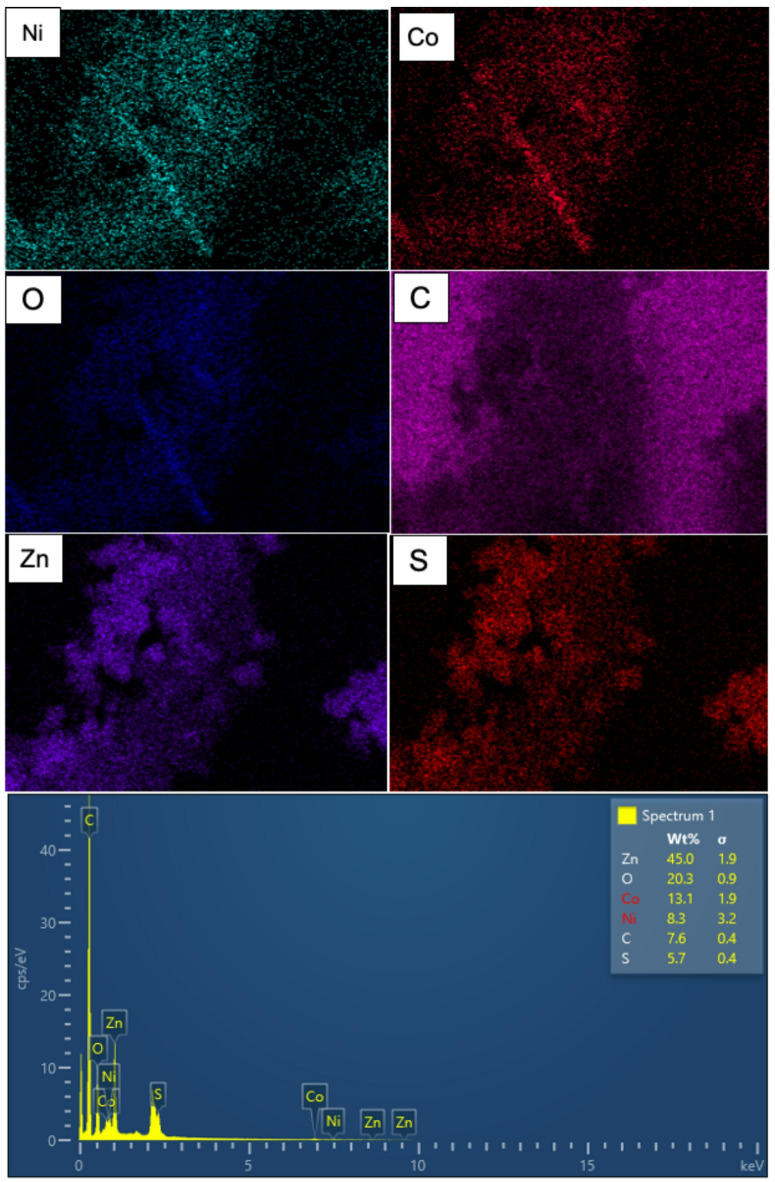
EDX spectra of the NCO@Z–MWCNT 9 wt% composite.

The EDX analysis verified the successful synthesis of the NCO@Z-MWCNT 9 wt% combination (Fig. 4). The survey spectra exclusively display the distinctive signals of Ni, Co, Zn, S, O, and C, signifying the absence of other metallic contaminants in the sample. Quantitative examination reveals the following weight percentages: Zn 45.0%, O 20.3%, Co 13.1%, Ni 8.3%, C 7.6%, and S 5.7%. The elevated concentrations of Ni, Co, and O are attributed to the spinel NiCo_2_O_4_ phase, while Zn and S derive from ZnS, and C is associated with the MWCNT support. The Ni/Co/O and Zn/S ratios align well with the nominal compositions of NiCo_2_O_4_ and ZnS, thereby corroborating the effective synthesis of the intended heterostructure.

The elemental mapping images exhibit a clearly delineated spatial distribution of each component. The signals of Ni and Co closely coincide with the O map, corroborating the establishment of NiCo_2_O_4_ as the primary oxide framework. The Zn and S signals are predominantly located in the same area, but have a slightly more diffuse appearance, indicating that ZnS is uniformly applied to the NiCo_2_O_4_ surface. Furthermore, the C map illustrates a uniform backdrop, signifying that the NiCo_2_O_4_@ZnS particles are consistently affixed to the MWCNT network. The EDX mapping and composition analysis confirm that NiCo_2_O_4_, ZnS, and MWCNT are closely integrated inside the NCO@Z-MWCNT composite.

**Fig. 5:**
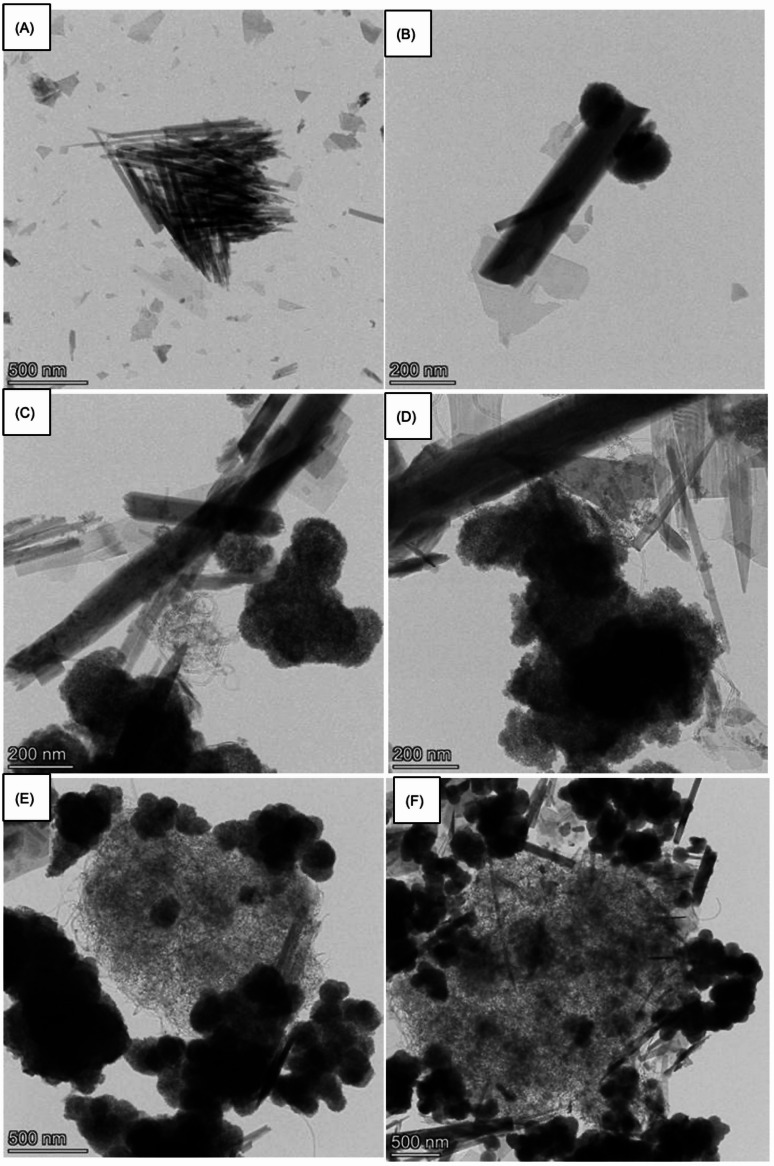
Representative TEM images of (**A**) Pure NCO; (**B**) NCO@Z; (**C**) NCO@Z-MWCNT 1 wt%; (**D**) NCO@Z-MWCNT 3 wt%; (**E**) NCO@Z-MWCNT 5 wt%; (**F**) NCO@Z-MWCNT 9 wt%.

The morphology and microstructure of the produced NCO as analyzed using TEM are illustrated in Fig. 5a. The TEM pictures indicate that the NCO nanoparticles consist in nanoneedles with an average particle size of around 500 nm. The nanoparticles exhibit uniform dispersion, showing limited agglomeration, which implies a substantial surface area and potential for improved electrochemical performance. In Fig. 5b, TEM images show the existence of black spherical formations, identifiable as ZnS. The ZnS nanoparticles are situated around the elongated and linear NiCo_2_O_4_ structures, indicating a robust interfacial contact between the two materials. The contrast variation observed in the TEM images arises from differences in electron density between ZnS and NiCo_2_O_4_ and confirms the coexistence of the two phases in the composite. This morphological configuration indicates that ZnS may have been either deposited on the NiCo_2_O_4_ surface or generated via a nucleation process during synthesis, potentially augmenting the composite’s electrochemical or catalytic characteristics, with an average particle size of around 200 nm. The NCO@Z-MWCNT 1 wt%, depicted in Fig. 5c, exhibits the lowest fluctuation in CNT composition. A notable worm-like structure, approximately 200 nm in size, is interwoven among the NiCo_2_O_4_@ZnS structures. This distinctive structural characteristic indicates the effective integration of CNTs, potentially leading to improved conductivity and structural integrity. The interwoven configuration of the CNTs with NiCo_2_O_4_@ZnS suggests robust interfacial contacts, likely enhancing electron transport and augmenting the material’s performance in applications like energy storage or catalysis. Additionally, as the MWCNT concentration rises to 3 wt%, 5 wt%, 7 wt%, and 9 wt%, a gradual development of worm-like structures is noted. This pattern is apparent in Fig. 5c–f, where the density and interconnectedness of these structures grow increasingly dramatic with elevation of MWCNT concentration.

### XPS analysis

**Fig. 6:**
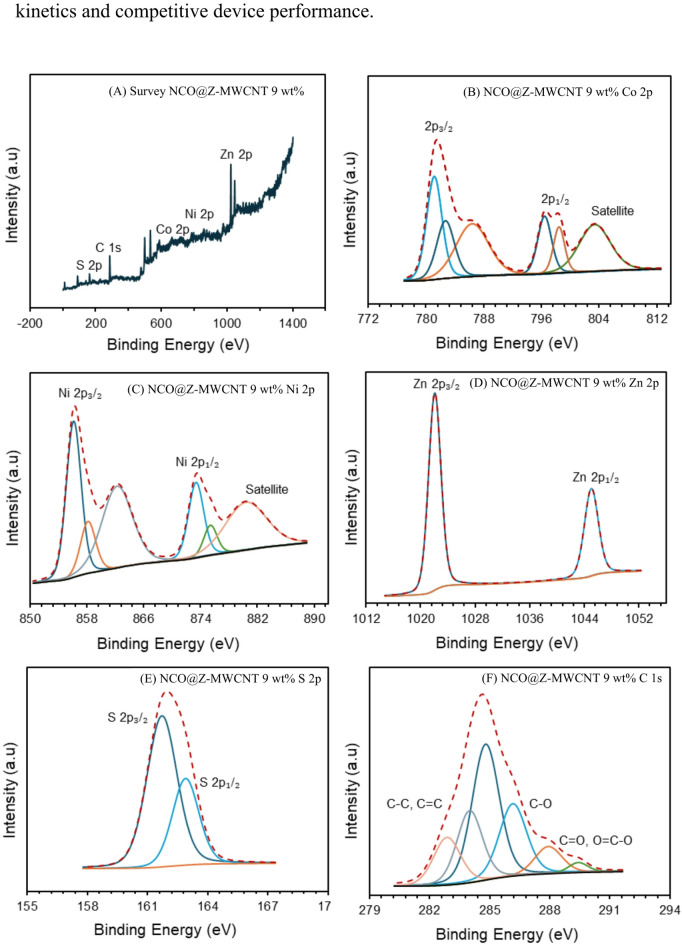
XPS spectra of the NCO@Z–MWCNT 9 wt% composite: (**A**) survey spectrum, (**B**) Co 2p, (**C**) Ni 2p, (**D**) Zn 2p, (**E**) S 2p and (**F**) C 1s.

Figure 6 presents the XPS spectra of the optimized NCO@Z–MWCNT 9 wt% composite, providing insight into its surface chemical composition and oxidation states, which are closely related to its electrochemical behaviour and device performance. The survey spectrum (Fig. 6a) confirms the presence of Ni, Co, Zn, S, O, and C without detectable impurities, indicating the successful integration of NiCo_2_O_4_, ZnS, and MWCNT components. The Co 2p spectrum (Fig. 6b) shows characteristic spin–orbit doublets of Co 2p_3_/_2_ and Co 2p_1_/_2_ together with satellite features, revealing the coexistence of Co^2+^ and Co^3+^ species typical of the spinel NiCo_2_O_4_ structure. Similarly, the Ni 2p spectrum (Fig. 6c) exhibits mixed Ni^2+^/Ni^3+^ valence states accompanied by shake-up satellites^22^. The coexistence of multiple oxidation states is generally associated with enhanced redox flexibility, which can facilitate charge-transfer reactions at the counter electrode–electrolyte interface. This behaviour is consistent with the reduced charge-transfer resistance observed in the EIS measurements and the improved fill factor obtained at the device level. The Zn 2p spectrum (Fig. 6d) displays two peaks at approximately 1021 eV and 1044 eV, corresponding to Zn 2p_3_/_2_ and Zn 2p_1_/_2_, respectively, confirming the presence of Zn^2+^ species derived from ZnS^23^. In addition, the S 2p region (Fig. 6e) shows two peaks at around 161.7 eV (S 2p_3_/_2_) and 162.9 eV (S 2p_1_/_2_), indicative of sulfide (S^2−^) species^23^. The absence of high-binding-energy features related to oxidized sulfur suggests that sulfur predominantly exists in the sulfide form, which is favorable for maintaining stable interfacial chemistry during repeated redox cycling. The C 1s spectrum (Fig. 6f) consists of three components at approximately 284.6 eV, 286.3 eV, and 288.6 eV, corresponding to C–C/C=C, C–O, and C=O/O–C=O bonds,^23,24^ respectively. The presence of oxygen-containing functional groups on the MWCNT surface can promote interfacial interactions between the oxide/sulfide phases and the carbon framework, contributing to improved electrical contact and continuous electron-transport pathways. This structural feature correlates well with the lower series resistance and charge-transfer resistance derived from EIS fitting, as well as the enhanced short-circuit current density and overall power conversion efficiency observed in DSSCs employing the NCO@Z–MWCNT 9 wt% counter electrode. Overall, the XPS results demonstrate the coexistence of mixed-valence NiCo_2_O_4_, ZnS, and a conductive MWCNT network, providing a coherent structure–property–performance relationship in which surface chemistry and interfacial interactions contribute to favorable charge-transfer.

### TGA analysis

**Fig. 7:**
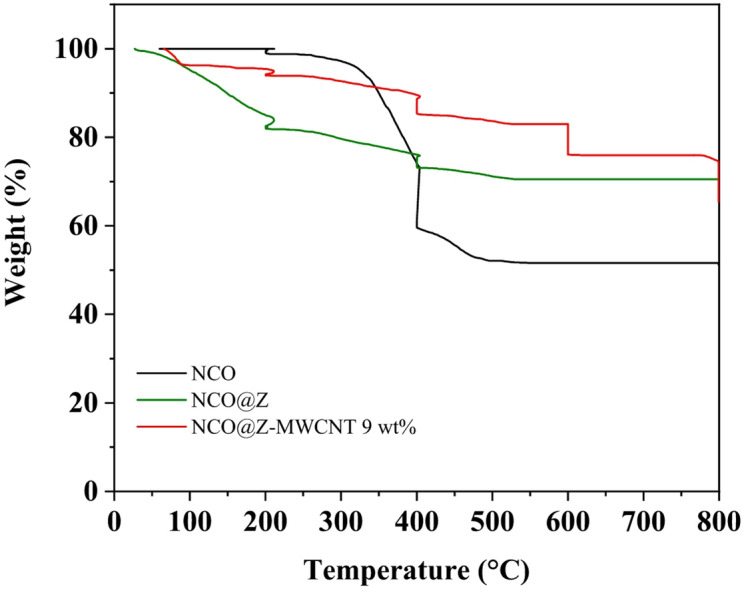
TGA profiles of pure NCO, NCO@Z, and NCO@Z–MWCNT 9 wt%, measured under an O_2_ atmosphere.

Figure 7 presents the thermogravimetric analysis (TGA) profiles of pure NCO, NCO@Z, and NCO@Z–MWCNT (9 wt%) measured under an O_2_ atmosphere at a heating rate of 20 °C min^−1^. For pure NCO (black curve), a minor weight loss of approximately 1.23% is observed in the temperature range of 200–250 °C, which can be attributed to the removal of physically adsorbed moisture and residual surface species^25^. A pronounced mass loss of about 39.18% occurs between 300 and 400 °C, which is associated with oxide decomposition and structural transformation of the spinel framework under oxidizing conditions, followed by an additional loss of 7.05% between 400 and 500 °C^26^ Above 500 °C, the remaining mass remains relatively constant up to 800 °C, indicating that the residual oxide framework exhibits limited further degradation at higher temperatures.

In the case of NCO@Z (green curve), a more pronounced initial weight loss of 14.68% below 150 °C is observed, which is attributed to the release of absorbed water and volatile surface residues introduced during the ZnS modification process. Subsequent weight losses of 3.2% (150–200 °C) and 5.76% (200–400 °C) arise from the oxidation and decomposition of sulfide-related surface species under an O_2_ atmosphere. After partial stabilization near 400 °C, additional mass losses of 2.92% and 2.57% are detected between 400 and 500 °C. Beyond this temperature, the residual mass remains nearly constant up to 800 °C. These results indicate that ZnS incorporation introduces additional thermally labile components in an oxidizing environment, rather than implying enhanced intrinsic phase stability at extreme temperatures.

By contrast, the NCO@Z–MWCNT (9 wt%) composite (red curve) exhibits a markedly reduced overall weight loss throughout the investigated temperature range. An initial loss of only 1.74% occurs below 200 °C, followed by gradual decreases of 2.09% between 200 and 400 °C and 1.68% around 400 °C, with only a minor additional loss of approximately 0.8% between 400 and 500 °C. Above this region, the mass remains nearly constant up to 800 °C. The significantly higher residual mass indicates improved thermal robustness of the hybrid composite relative to pure NCO and NCO@Z under identical oxidizing conditions. This enhanced robustness is attributed to the stabilizing effect of the MWCNT network, which helps maintain structural integrity and suppress rapid mass loss during high-temperature oxidation, rather than indicating absolute thermal stability of individual components^11,27^.

The TGA profiles of the NCO@Z–MWCNT composites with 3 wt%, 5 wt%, and 7 wt% MWCNT are provided in Fig. S4. Thermogravimetric analyses were performed for NCO, NCO@Z, and NCO@Z–MWCNT composites with 3 wt%, 5 wt%, 7 wt%, and 9 wt% MWCNT loadings. The residual masses at 800 °C were determined to be 51.19% for NCO, 70.52% for NCO@Z, and 88.11%, 89.97%, 91.90%, and 65.44% for NCO@Z–MWCNT composites with 3 wt%, 5 wt%, 7 wt%, and 9 wt% MWCNT, respectively.

The higher residue values observed for the 3–7 wt% composites originate from the dominance of thermally stable metal oxide/sulfide frameworks (NiCo_2_O_4_ and Zn-containing phases) remaining after oxidative decomposition of MWCNTs, whereas the comparatively lower residue of the 9 wt% MWCNT sample is attributed to the increased fraction of oxidizable carbon undergoing combustion at elevated temperatures under an O_2_ atmosphere.

It should be emphasized that the TGA measurements were conducted under an oxygen atmosphere, where oxidative degradation of sulfide and carbonaceous components is expected at elevated temperatures. Accordingly, the observed mass losses at high temperatures reflect oxidative processes rather than intrinsic instability under practical operating conditions. Within the temperature range relevant to DSSC operation, the NCO@Z–MWCNT composite exhibits sufficient thermal stability. The reduced overall mass loss compared with NCO and NCO@Z indicates that incorporation of MWCNTs contributes to improved structural integrity of the hybrid system, supporting its suitability for practical counter-electrode applications, without implying stability under extreme high-temperature oxidative environments^21^.

### BET analysis

To evaluate the textural properties and porosity of the synthesized materials, nitrogen adsorption–desorption measurements were performed on pure NCO, NCO@Z, and NCO@Z–MWCNT 9 wt%. The Brunauer–Emmett–Teller (BET) surface areas were determined to be 7.85, 10.25, and 8.24 m^2^ g^−1^, respectively. The increase in surface area following ZnS incorporation suggests the introduction of additional accessible surface features, whereas the slight decrease observed for the NCO@Z–MWCNT composite can be attributed to partial pore coverage or aggregation associated with the incorporation of the MWCNT network^11^.

**Fig. 8:**
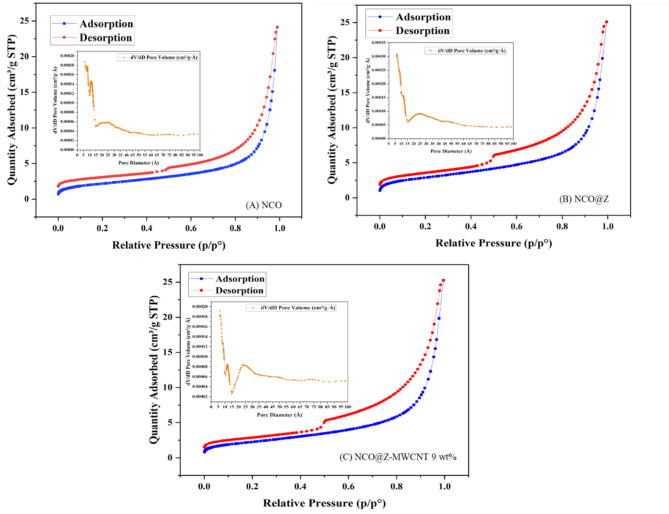
BET analysis of N_2_ adsorption–desorption isotherms and (inset) BJH pore-size distribution plot of (**A**) Pure NCO, (**B**) NCO@Z, and (**C**) NCO@Z-MWCNT 9 wt%.

The pore size distributions derived from the Barrett–Joyner–Halenda (BJH) method indicate that all samples possess average pore diameters in the range of 5–10 nm, confirming their mesoporous nature. The nitrogen adsorption–desorption isotherms of pure NCO (Fig. 8a), NCO@Z (Fig. 8b), and NCO@Z–MWCNT 9 wt% (Fig. 8c) exhibit typical Type IV behavior with pronounced hysteresis loops, characteristic of mesoporous structures formed via capillary condensation^28^. From an electrochemical perspective, the presence of mesoporosity combined with moderate surface area is favorable for counter electrode operation, as it can facilitate electrolyte infiltration and ion transport while providing a sufficient density of electrochemically accessible sites. These textural features are consistent with the reduced charge-transfer resistance observed in EIS measurements and the improved current density and fill factor at the device level. Accordingly, the BET and BJH results support a structure–property–performance relationship in which optimized porosity and surface characteristics contribute to the enhanced electrocatalytic behavior of the NCO@Z–MWCNT composites in DSSCs.

### The photocurrent density-voltage (J–V) curves

**Fig. 9:**
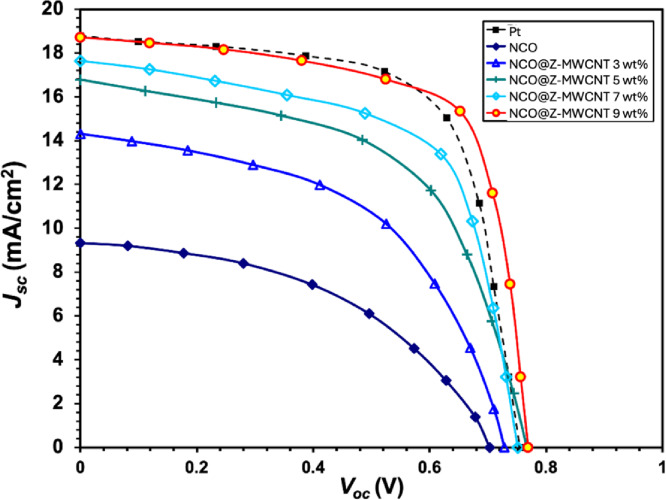
J–V curves of DSSCs based on Pt, NCO, and NCO@Z-MWCNT 3 wt%, 5 wt%, 7 wt%, and 9 wt%.

Figure 9 illustrates the current density–voltage (J–V) characteristics of dye-sensitized solar cells (DSSCs) constructed with Pt, pure NCO, NCO@Z, and NCO@ZnS–MWCNT composites, with varying MWCNT loadings of 3 wt%, 5 wt%, 7 wt%, and 9 wt% as counter electrodes. The corresponding photovoltaic parameters, including the open-circuit voltage (V_OC_), short-circuit current density (J_SC_), fill factor (FF), and power conversion efficiency (η), are summarized in Table 1. The J–V measurements were conducted to assess the photovoltaic performance and electrocatalytic efficacy of the produced electrodes relative to the traditional Pt electrode. The pure NCO counter electrode exhibits suboptimal performance (3.1%) due to its comparatively low electrical conductivity and elevated charge-transfer resistance at the electrolyte–electrode contact. The device performance improved upon modification with ZnS, which was attributable to greater interfacial characteristics and superior charge separation resulting from the creation of a heterojunction between NiCo_2_O_4_ and ZnS. A more significant improvement is noted with the inclusion of MWCNTs. As the MWCNT concentration is increases from 3% to 9%, both the short-circuit current density (*J*_*SC*_) and open-circuit voltage (*V*_*OC*_) exhibit a consistent enhancement (Table 1). This tendency is ascribed to the superior electrical conductivity, elevated aspect ratio, and rapid electron transport channels afforded by the MWCNT network, which markedly diminish series resistance and enhance electron collection. Of all the evaluated electrodes, the NCO@Z–MWCNT 9 wt% sample demonstrates the best photovoltaic performance (10.03%), exceeding that of the traditional Pt electrode (9.6%), as shown in Table 1. The enhanced performance can be attributed to the combined contributions of the catalytically active NiCo_2_O_4_ phase, the presence of ZnS associated with the NiCo_2_O_4_ surface, and the conductive MWCNT framework. Together, these components provide favorable charge-transfer pathways and increased electrochemically active sites, thereby promoting catalytic activity toward the I^−^/I_3_^−^ redox couple^29^. It is important to recognize that while increased MWCNT concentrations enhance conductivity, excessive amounts may result in nanotube aggregation or partial obstruction of light absorption, thereby impairing device performance. In this work, a 9 wt% MWCNT composition is identified as best, achieving a balance of electrical conductivity, catalytic activity, and optical clarity. The findings indicate that the NCO@Z–MWCNT 9 wt% composite is a highly promising, cost-effective substitute for platinum counter electrodes in high-performance dye-sensitized solar cells^11,23^.

**Table 1:** DSSCs Performance Using different electrode composition.

CEs	Voc (V)	Jsc (mA/cm^2^)	Fill factor	Efficiency (η) %	Est. performance trend	Standard deviation
Pt	0.74	17.5	0.67	9.6	High	± 0.099
NCO	0.65	9.0	0.47	3.1	Low	± 0.124
NCO@Z-MWCNT 3 wt%	0.68	12.0	0.51	5.4	Moderate	± 0.119
NCO@Z-MWCNT 5 wt%	0.72	15.5	0.56	7.2	High	± 0.122
NCO@Z-MWCNT 7 wt%	0.75	17.0	0.63	8.3	Higher	± 0.121
NCO@Z-MWCNT 9 wt%	0.78	19.5	0.70	10.03	Highest	± 0.116

### Cyclic voltammetry analysis

**Fig. 10:**
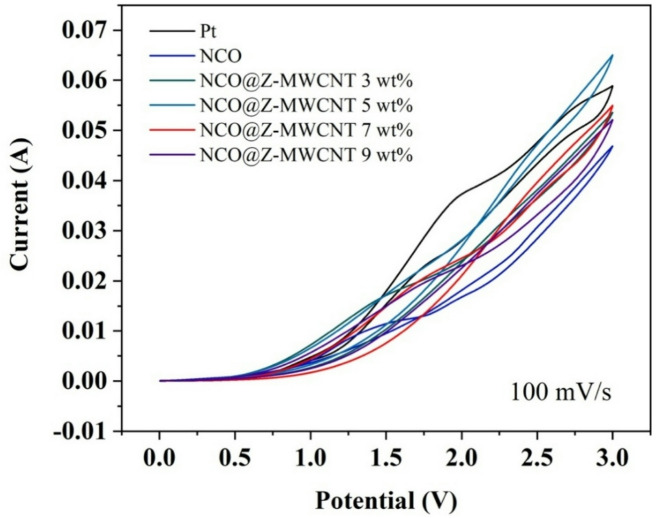
Cyclic voltammetry at 100 mV s^−1^ of Pt, NCO, and NCO@Z-MWCNT 3 wt%, 5 wt%, 7 wt%, and 9 wt%.

Figure 10 presents the cyclic voltammetry (CV) curves of Pt, pure NiCo_2_O_4_, and NCO@Z–MWCNT counter electrodes with different MWCNT loadings (3 wt%, 5 wt%, 7 wt%, and 9 wt%) recorded at a scan rate of 100 mV s^−^¹. As the electrode composition evolves from bare NCO to ZnS and MWCNT-modified composites, a clear enhancement in both anodic and cathodic currents is observed. This improvement reflects accelerated redox kinetics for the I^−^/I_3_^−^ redox couple. The NCO@Z–MWCNT 9 wt% electrode, among all compositions, demonstrates the best electrochemical performance, providing an optimal conductive network formation. The electrode exhibits the largest current response and a smaller peak-to-peak separation, indicating faster electron transfer and lower polarization. In contrast, bare NiCo_2_O_4_ shows the lowest redox current and the largest onset overpotential, confirming its inferior catalytic activity. The incorporation of ZnS introduces additional sulfide active sites, while the MWCNT network provides highly conductive pathways, enabling efficient electron transport throughout the electrode. As a result, the current density across the entire potential window increases, and the onset potential shifts toward lower overpotential. Notably, the 9 wt% composite delivers a current response approaching or even exceeding that of the Pt electrode at higher potentials.

### EIS analysis

**Fig. 11:**
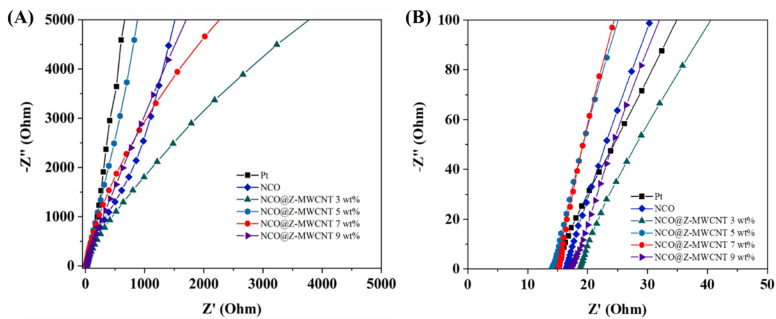
Electrochemical characterization of Pt and NCO based counter electrodes. (**a**) Nyquist plots (Z′ vs. − Z″) and (**b**) zoomed-in view of symmetric cells measured in the I^−^/I_3_^−^ electrolyte at room temperature for Pt, NCO, and NCO@Z-MWCNT 3 wt%, 5 wt%, 7 wt%, and 9 wt%.

Electrochemical impedance spectroscopy (EIS) was employed to investigate the interfacial charge-transfer behaviour and electrocatalytic properties of the prepared counter electrodes, as shown in Fig. 11. The fitted EIS parameters, obtained using the equivalent circuit presented in Fig. S5^30^—including the series resistance (R_s_), charge-transfer resistance (R_ct_), resistance associated with the counter-electrode film (R_rce_), and constant phase elements (CPEs)—are summarized in Table 2. The R_s_ values for all electrodes fall within a comparable range, indicating that the ohmic contributions from the FTO substrate, electrolyte, and electrical contacts are essentially similar. This suggests that the observed performance differences predominantly arise from interfacial charge-transfer processes rather than bulk resistance effects.

In contrast, substantial variations in R_ct_ are observed among the electrodes. The pristine NCO electrode exhibits a relatively high R_ct_, indicating sluggish charge-transfer kinetics toward the reduction of triiodide (I_3_^−^). Upon incorporation of ZnS and MWCNTs, the R_ct_ values decrease markedly, reflecting enhanced interfacial charge-transfer behavior at the counter electrode/electrolyte interface. In particular, the NCO@Z–MWCNT electrodes with higher MWCNT contents display progressively lower R_ct_ values. This improvement is associated with surface modification by ZnS and the formation of an interconnected conductive network provided by the MWCNTs, which together facilitate more efficient electron transport and interfacial charge exchange.

The enhanced electrochemical behaviour, evidenced by increased current responses in cyclic voltammetry and reduced charge-transfer resistance, correlates well with the photovoltaic performance of the corresponding DSSCs. Specifically, the lower R_ct_ and improved redox kinetics are accompanied by increases in short-circuit current density (J_SC_) and fill factor, leading to the highest power conversion efficiency of 10.03% for the device employing the NCO@Z–MWCNT (9 wt%) counter electrode. Overall, these results demonstrate that the optimized integration of ZnS and MWCNTs with NiCo_2_O_4_ effectively improves interfacial charge transport and electrocatalytic behavior, rendering the NCO@Z–MWCNT (9 wt%) composite a competitive Pt-free counter-electrode candidate for dye-sensitized solar cell applications.

**Table 2:** Fitted EIS parameters of Pt, NCO, and NCO@Z-MWCNT 3 wt%, 5 wt%, 7 wt%, and 9 wt%.

Samples	*R*_s_ (Ω)	*R*_ct_ (Ω)	*R*_rce_ (Ω)	CPE-1		CPE-2	
				CPE-T (×10^− 5^)	CPE-*P*	CPE-T (×10^− 5^)	CPE-*P*
Pt	14.967	306.44	130,140	9.226	0.813	1.776	0.981
NCO	16.555	987.24	94,689	2.490	0.914	1.958	0.959
NCO@Z-MWCNT 3 wt%	20.145	758.25	259,480	9.201	0.832	1.984	0.980
NCO@Z-MWCNT 5 wt%	14.159	502.98	240,480	6.728	0.892	1.850	0.974
NCO@Z-MWCNT 7 wt%	14.469	331.75	229,840	8.884	0.861	1.893	0.968
NCO@Z-MWCNT 9 wt%	17.227	238.23	149,380	4.431	1.095	3.097	0.869

## Conclusion

NCO nanorods were integrated with ZnS and an MWCNT network to construct a Pt-free counter electrode for DSSCs using a hierarchical design approach. XRD confirmed the coexistence of cubic NiCo_2_O_4_ and zinc blende ZnS phases. FESEM combined with EDS showed that ZnS was distributed on the surfaces of the nanorods, while the MWCNTs formed a continuous conductive framework bridging adjacent rods. XPS analysis identified mixed-valence Ni^2+^/Ni^3+^ and Co^2+^/Co^3+^ species characteristic of the spinel structure, together with Zn^2+^ and S^2−^, consistent with the incorporation of ZnS. Thermogravimetric analysis indicated that the NCO@Z–MWCNT 9 wt% composite exhibited the lowest overall mass loss, suggesting improved thermal stability.

Electrochemical measurements revealed the cumulative contributions of each component. Cyclic voltammetry showed increased redox current density and a reduced onset potential when progressing from NCO to NCO@Z and further to NCO@Z–MWCNT composites with increasing MWCNT content. Consistently, Nyquist plots exhibited a reduced high-frequency intercept and a smaller semicircle diameter, corresponding to lower series resistance and charge-transfer resistance at the counter electrode–electrolyte interface. These electrochemical trends were reflected at the device level, where DSSCs employing NCO@Z–MWCNT 9 wt% as the counter electrode achieved a power conversion efficiency of 10.03%, slightly exceeding that of the Pt reference electrode (9.6%), along with enhancements in short-circuit current density and fill factor.

Rather than implying a fundamentally new mechanism, the results indicate that the components in the optimized combination perform complementary roles. The presence of ZnS is associated with surface modification and interfacial effects on the oxide nanorods, while the MWCNT network facilitates efficient electron transport and improved electrical contact with the current collector. Together, this hierarchical architecture supports effective triiodide reduction and mitigates resistive losses. Owing to its compatibility with low-temperature solution processing and stability in iodide/triiodide electrolytes, this work demonstrates a practical and scalable strategy for developing competitive Pt-free counter electrodes for dye-sensitized solar cells.

## Supplementary Information

Below is the link to the electronic supplementary material.

Supplementary Material 1

## Data Availability

The datasets generated and analyzed during the current study are available within the article information files. Raw data supporting the conclusions of this article are available from the corresponding author upon reasonable request.
