# Supplementary material for: Remodelling hierarchical NiCo2O4@ZnS nanorods with multi-walled carbon nanotubes as a counter electrode for dye-sensitized solar cell applications
Source: Sci Rep. 2026 Feb 1;16:6869. doi: 10.1038/s41598-026-38255-7 (PMC12916951; doi:10.1038/s41598-026-38255-7)
Supplement: Supplementary file 1 — Supplementary Material 1 [file 41598_2026_38255_MOESM1_ESM.docx]

**Supplementary Information**

**Remodelling Hierarchical NiCo_2_O_4_@ZnS Nanorods with the Multi-Walled Carbon Nanotube as Counter electrode for Dye-sensitized Solar cells Application**

***Methawee Nukunudompanich^a^, Theeranuch Nachaithong^b^, Phatcharin Phumuen^c^, Wassana Wannabut^c^, Neeraphat Kunbuala^d^***, ***Supinya Nijpanich^e^, Kongsak Pattarith^f^, and Yonrapach Areerob^a*^***

1. *Department of Industrial Engineering, School of Engineering, King Mongkut’s Institute of Technology Ladkrabang, Bangkok 10520, Thailand*
2. *Institute for Integrated Radiation and Nuclear Science, Kyoto University, 2-1010, Asahiro Nishi, Kumatori, Sennan-gun, Osaka, 590-0494, Japan*
3. *Department of Physics, Faculty of Science, Khon Kaen University, Khon Kaen, 40002, Thailand*
4. *Department of Biomedical Engineering, Tohoku University, Sendai 980-8579, Japan*
5. *Synchrotron Light Research Institute (Public Organization), 111 University Avenue, Muang District, Nakhon Ratchasima 30000, Thailand*
6. *Department of Chemistry, Faculty of Science, Buriram Rajabhat University, Buriram 31000, Thailand*

*Corresponding Email;* [*yonrapach.ar@kmitl.ac.th*](mailto:yonrapach.ar@kmitl.ac.th) *Tel:+66 964245359*


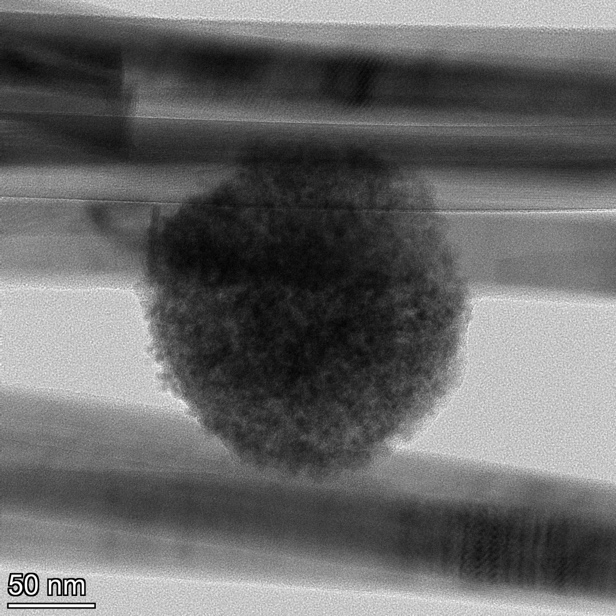


**Supplementary Figure 1.** TEM image showing a nanosphere-like NiCo₂O₄ aggregate composed of densely packed polycrystalline nanoparticles, attached to the one-dimensional backbone, forming a hierarchical structure.


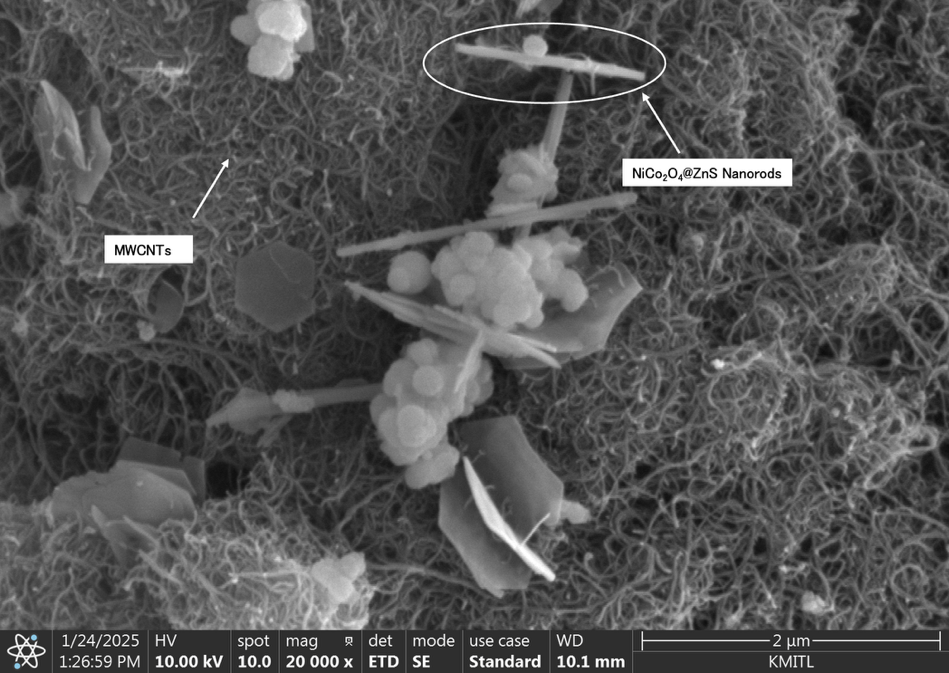


**(A)**


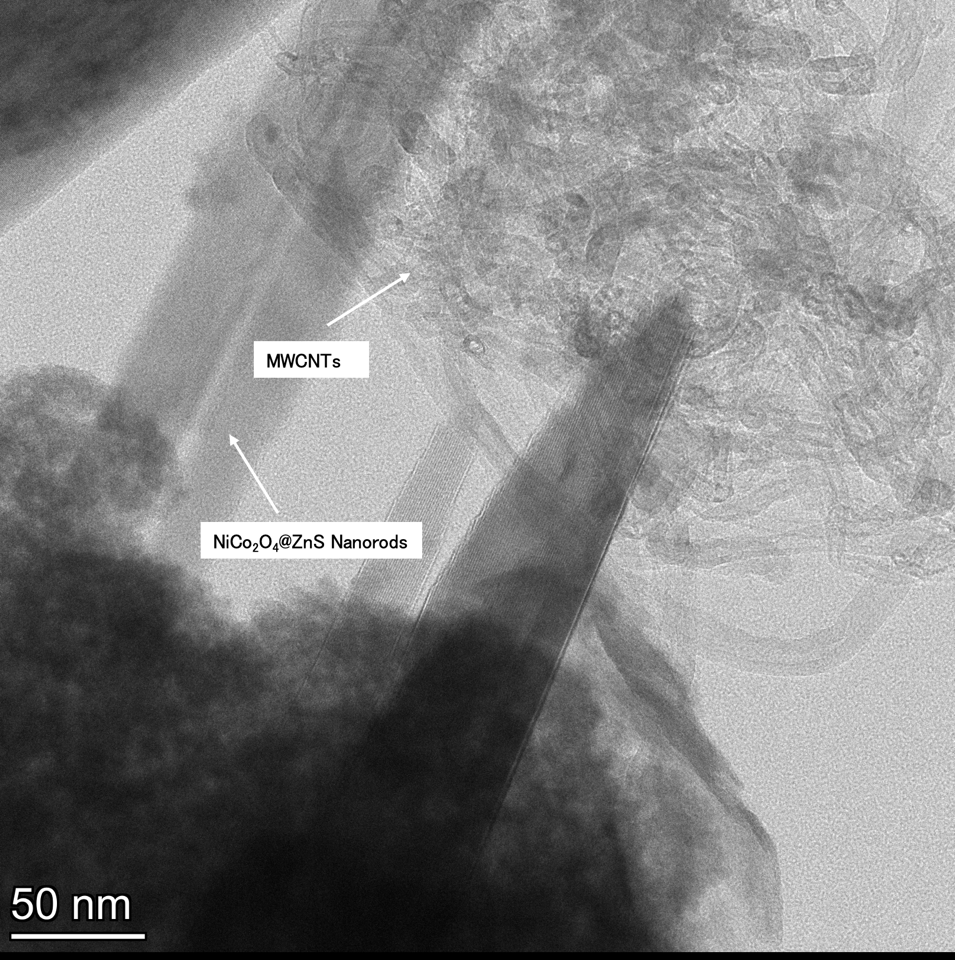


**(B)**

**Supplementary Figure S2.** (A) High-magnification FESEM image and (B) TEM image of the NCO@Z–MWCNT (9) composite. The fibrous, thread-like structures highlighted by arrows are attributed to MWCNTs, which form an interconnected conductive network surrounding and bridging the NiCo₂O₄@ZnS nanorods. These observations provide direct morphological evidence supporting the incorporation of MWCNTs into the composite structure.

**Supplementary Figure S3.** J–V characteristics of the dye-sensitized solar cell employing **MWCNTs alone as the counter electrode** under simulated AM 1.5G illumination (100 mW/cm²). The inset summarizes the corresponding photovoltaic parameters, including the open-circuit voltage ($V_{\text{oc}}$), short-circuit current density ($J_{\text{sc}}$), fill factor (FF), and power conversion efficiency (PCE), serving as a reference for comparison with the composite counter electrodes discussed in the main text.

**Supplementary Figure S4.** Thermogravimetric analysis (TGA) curves of NCO, NCO@Z, and NCO@Z–MWCNT composites with MWCNT loadings of 3, 5, 7, and 9 wt%, measured under an O₂ atmosphere. The residual masses at 800 °C are 51.19% for NCO, 70.52% for NCO@Z, and 88.11%, 89.97%, 91.90%, and 65.44% for NCO@Z–MWCNT composites with 3, 5, 7, and 9 wt% MWCNT, respectively. The reduced residual mass observed at higher MWCNT loading (9 wt%) is attributed to enhanced oxidative decomposition of the carbon component under an oxygen atmosphere.


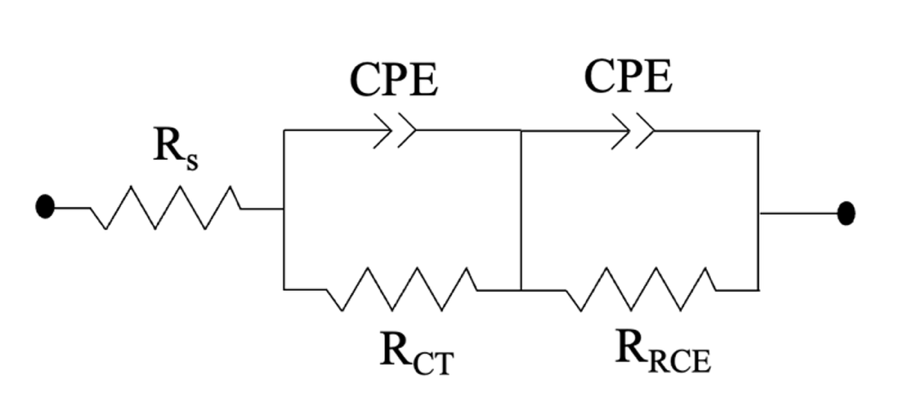


**Supplementary Figure S5.** The equivalent circuit model used to fit the EIS spectra of the symmetric dummy cell (CE‖CE) consists of a series resistance (R_s_) connected to two parallel combinations of constant phase elements (CPEs) and resistances. In this model, R_s_ represents the overall series resistance of the system. The first parallel branch, composed of R_ct_ and a CPE, corresponds to the charge-transfer process at the counter electrode/electrolyte interface, while the second parallel branch, consisting of R_rce_ and a CPE, accounts for the resistance associated with the counter-electrode film and secondary interfacial processes. The CPEs are introduced to describe the non-ideal capacitive behavior arising from surface roughness and interfacial heterogeneity.
